# Supplementary material for: An in silico approach to study the role of epitope order in the multi-epitope-based peptide (MEBP) vaccine design
Source: Sci Rep. 2022 Jul 22;12:12584. doi: 10.1038/s41598-022-16445-3 (PMC9307121; doi:10.1038/s41598-022-16445-3)
Supplement: Supplementary file 1 — Supplementary Information 1. [file 41598_2022_16445_MOESM1_ESM.docx]

**An *in silico* approach to study the role of epitope order in the multi-epitope-based peptide (MEBP) vaccine design**

**Muthu Raj Salaikumaran^1^, Prasanna Sudharson Kasamuthu^1^, Veeranarayanan Surya Aathmanathan^1^, Burra V L S Prasad^1^***

^1^Centre for Advanced Research and Innovation in Structural Biology of Diseases, K L E F University, Vaddeswaram, Andhra Pradesh 522 502, India

^*^Corresponding Author: [dr.prasad.bvls@gmail.com](mailto:dr.prasad.bvls@gmail.com)

**Supplementary Material 1**

**MEBP Variants in FASTA format**

>REF_SEQ

### GIINTLQKYYCRVRGGRCAVLSCLPKEEQIGKCSTRGRKCCRRKKEAAAKTLDSKTQSLAAYGKQGNFKNLAAYCYGV

### SPTKLAAYKIADYNYKLAAYVVVLSFELLGPGPGIGINITRFQGPGPGYGFQPTNGVGPGPGVLSFELLHAGPGPGLQ

### IPFAMQMGPGPGIAIVMVTIMHHHHHH

### >SPVC_206

### GIINTLQKYYCRVRGGRCAVLSCLPKEEQIGKCSTRGRKCCRRKKEAAAKYGFQPTNGVGPGPGGKQGNFKNLAAYVL

### SFELLHAGPGPGIGINITRFQGPGPGKIADYNYKLAAYTLDSKTQSLAAYCYGVSPTKLAAYIAIVMVTIMGPGPGVV

### VLSFELLGPGPGLQIPFAMQMHHHHHH

### >SPVC_214

### GIINTLQKYYCRVRGGRCAVLSCLPKEEQIGKCSTRGRKCCRRKKEAAAKVLSFELLHAGPGPGGKQGNFKNLAAYCY

### GVSPTKLAAYKIADYNYKLAAYTLDSKTQSLAAYYGFQPTNGVGPGPGVVVLSFELLGPGPGIAIVMVTIMGPGPGIG

### INITRFQGPGPGLQIPFAMQMHHHHHH

### >SPVC_32

### GIINTLQKYYCRVRGGRCAVLSCLPKEEQIGKCSTRGRKCCRRKKEAAAKCYGVSPTKLAAYKIADYNYKLAAYTLDS

### KTQSLAAYYGFQPTNGVGPGPGGKQGNFKNLAAYIGINITRFQGPGPGVVVLSFELLGPGPGLQIPFAMQMGPGPGIA

### IVMVTIMGPGPGVLSFELLHAHHHHHH

### >SPVC_357

### GIINTLQKYYCRVRGGRCAVLSCLPKEEQIGKCSTRGRKCCRRKKEAAAKTLDSKTQSLAAYYGFQPTNGVGPGPGCY

### GVSPTKLAAYVVVLSFELLGPGPGIAIVMVTIMGPGPGIGINITRFQGPGPGKIADYNYKLAAYGKQGNFKNLAAYVL

### SFELLHAGPGPGLQIPFAMQMHHHHHH

### >SPVC_537

### GIINTLQKYYCRVRGGRCAVLSCLPKEEQIGKCSTRGRKCCRRKKEAAAKCYGVSPTKLAAYKIADYNYKLAAYGKQG

### NFKNLAAYYGFQPTNGVGPGPGVVVLSFELLGPGPGLQIPFAMQMGPGPGIAIVMVTIMGPGPGTLDSKTQSLAAYIG

### INITRFQGPGPGVLSFELLHAHHHHHH

### >SPVC_383

### GIINTLQKYYCRVRGGRCAVLSCLPKEEQIGKCSTRGRKCCRRKKEAAAKKIADYNYKLAAYGKQGNFKNLAAYVVVL

### SFELLGPGPGTLDSKTQSLAAYCYGVSPTKLAAYYGFQPTNGVGPGPGVLSFELLHAGPGPGIAIVMVTIMGPGPGIG

### INITRFQGPGPGLQIPFAMQMHHHHHH

### >SPVC_565

### GIINTLQKYYCRVRGGRCAVLSCLPKEEQIGKCSTRGRKCCRRKKEAAAKCYGVSPTKLAAYKIADYNYKLAAYYGFQ

### PTNGVGPGPGVVVLSFELLGPGPGTLDSKTQSLAAYIGINITRFQGPGPGIAIVMVTIMGPGPGLQIPFAMQMGPGPG

### GKQGNFKNLAAYVLSFELLHAHHHHHH

### >SPVC_446

### GIINTLQKYYCRVRGGRCAVLSCLPKEEQIGKCSTRGRKCCRRKKEAAAKYGFQPTNGVGPGPGVVVLSFELLGPGPG

### GKQGNFKNLAAYIAIVMVTIMGPGPGCYGVSPTKLAAYTLDSKTQSLAAYLQIPFAMQMGPGPGVLSFELLHAGPGPG

### KIADYNYKLAAYIGINITRFQHHHHHH

### >SPVC_387

### GIINTLQKYYCRVRGGRCAVLSCLPKEEQIGKCSTRGRKCCRRKKEAAAKGKQGNFKNLAAYVLSFELLHAGPGPGIG

### INITRFQGPGPGVVVLSFELLGPGPGCYGVSPTKLAAYTLDSKTQSLAAYKIADYNYKLAAYYGFQPTNGVGPGPGLQ

### IPFAMQMGPGPGIAIVMVTIMHHHHHH
