# Supplementary material for: An in silico approach to study the role of epitope order in the multi-epitope-based peptide (MEBP) vaccine design
Source: Sci Rep. 2022 Jul 22;12:12584. doi: 10.1038/s41598-022-16445-3 (PMC9307121; doi:10.1038/s41598-022-16445-3)
Supplement: Supplementary file 2 — Supplementary Figures. [file 41598_2022_16445_MOESM2_ESM.docx]

**An *in silico* approach to study the role of epitope order in the multi-epitope-based peptide (MEBP) vaccine design**

**Muthu Raj Salaikumaran^1^, Prasanna Sudharson Kasamuthu^1^, Veeranarayanan Surya Aathmanathan^1^, Burra V L S Prasad^1^***

^1^Centre for Advanced Research and Innovation in Structural Biology of Diseases, K L E F University, Vaddeswaram, Andhra Pradesh 522 502, India

^*^Corresponding Author: [dr.prasad.bvls@gmail.com](mailto:dr.prasad.bvls@gmail.com)

**Supplementary Material 2**

**C-Immsim results**

**A B**


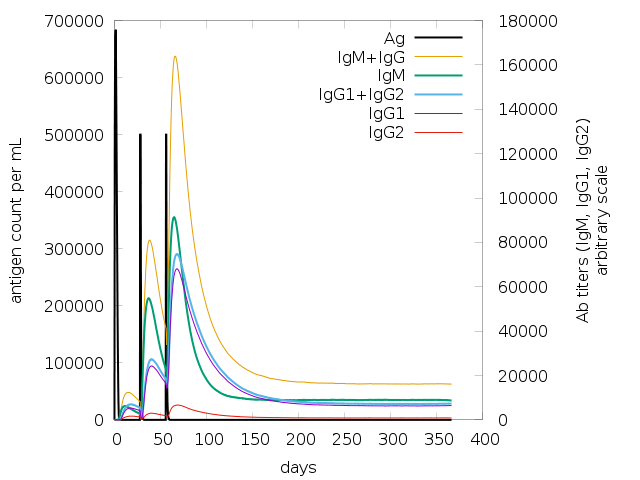

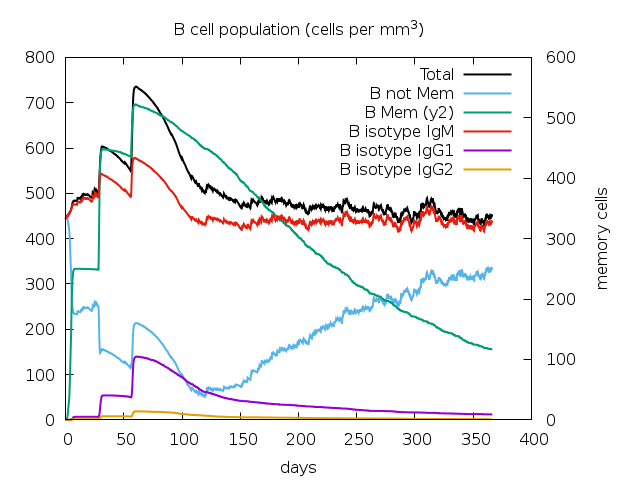


**C D**


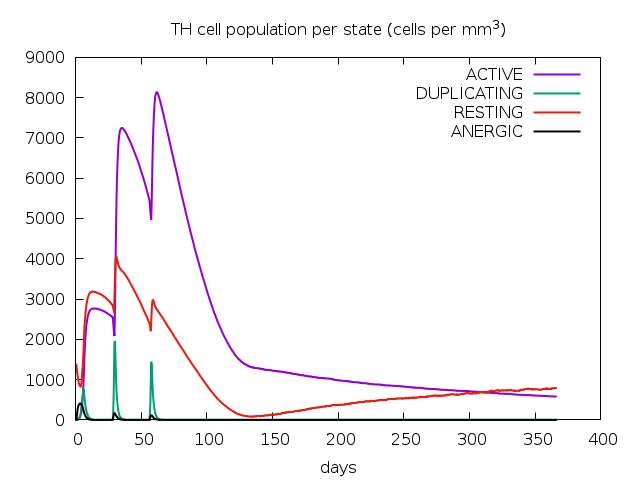

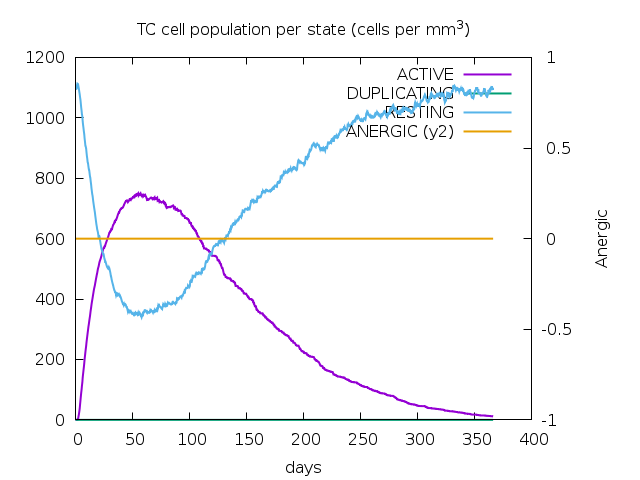


**Figure S1: SPVC_214 with adjuvants+HIS-tag:A)Antigen and immunoglobulin counts B)The changes observed in B-cell populations after given three injections, C) The development of T-helper, and D) T-cytotoxic cell populations per state after the injections**

**A B**

**
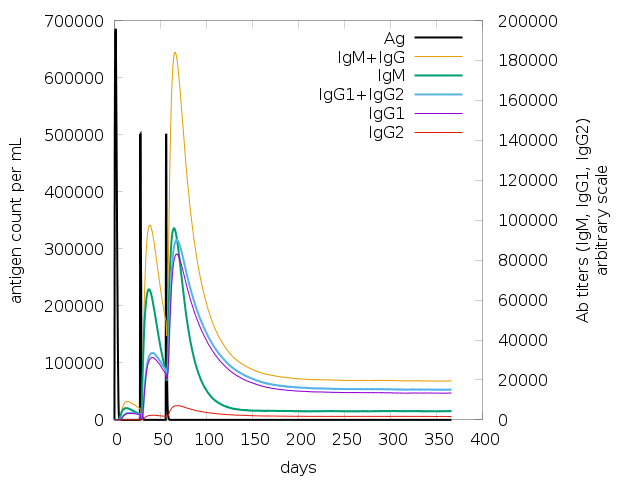

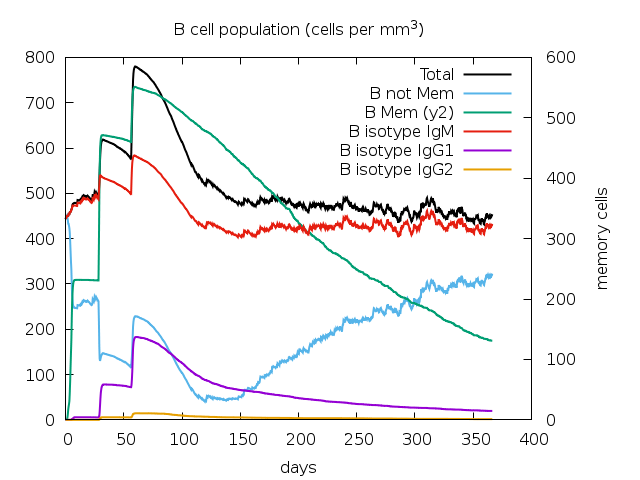
**

**C D**

**
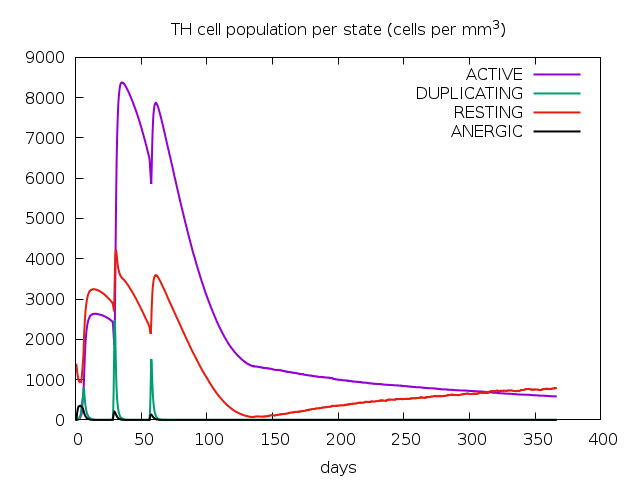

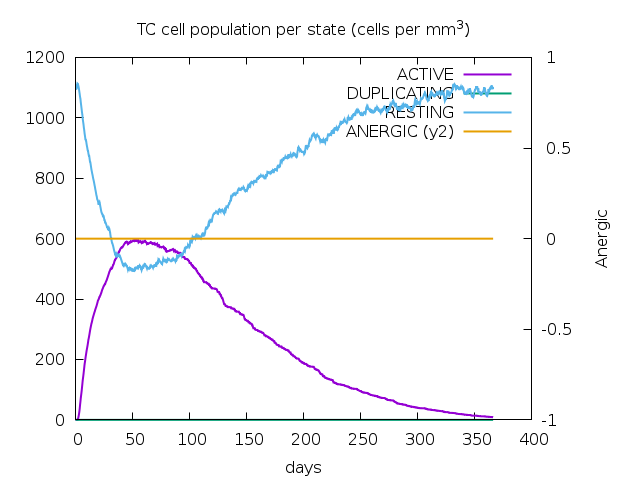
**

**Figure S2: SPVC_32 with adjuvants+HIS-tag:A)Antigen and immunoglobulin counts B)The changes observed in B-cell populations after given three injections, C) The development of T-helper, and D) T-cytotoxic cell populations per state after the injections**

**A B**


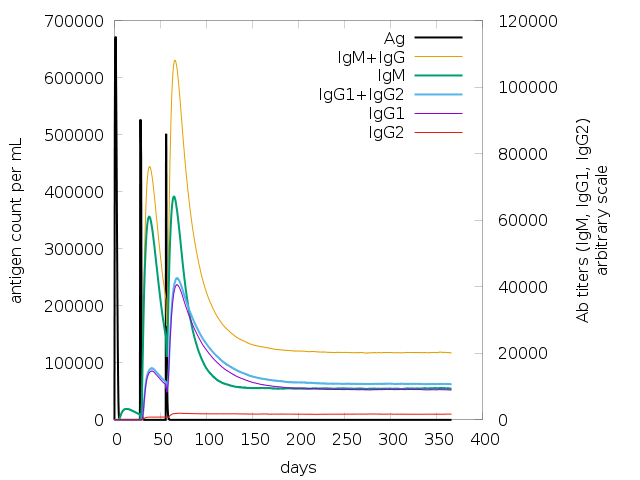

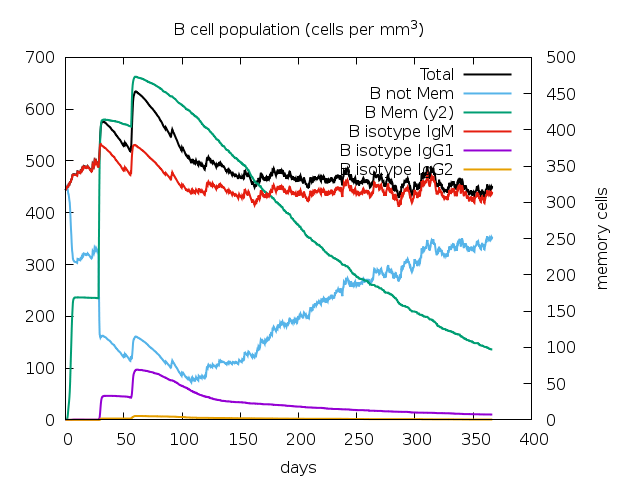


**C D**


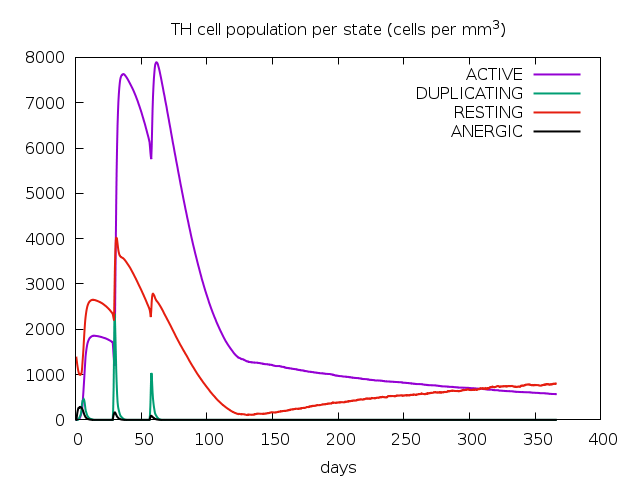

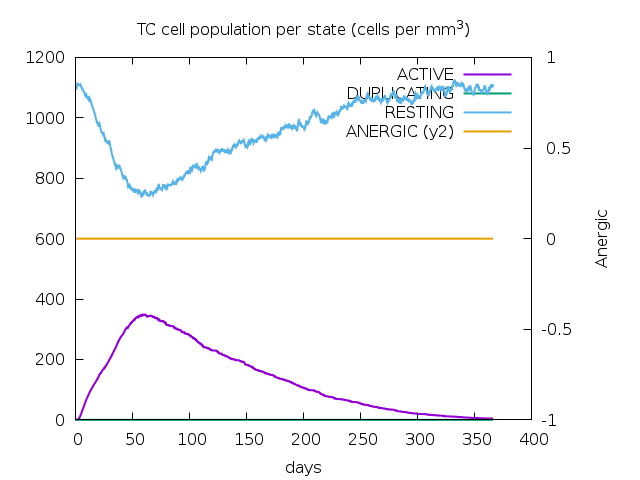


**Figure S3: SPVC_206 with adjuvants+HIS-tag:A)Antigen and immunoglobulin counts B)The changes observed in B-cell populations after given three injections, C) The development of T-helper, and D) T-cytotoxic cell populations per state after the injections**

**A B**

**
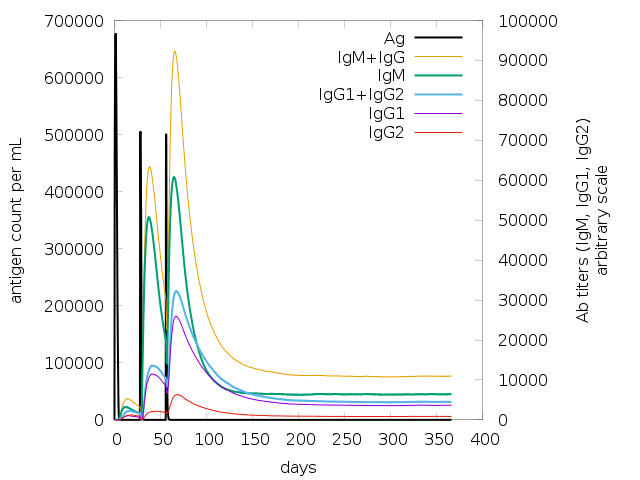

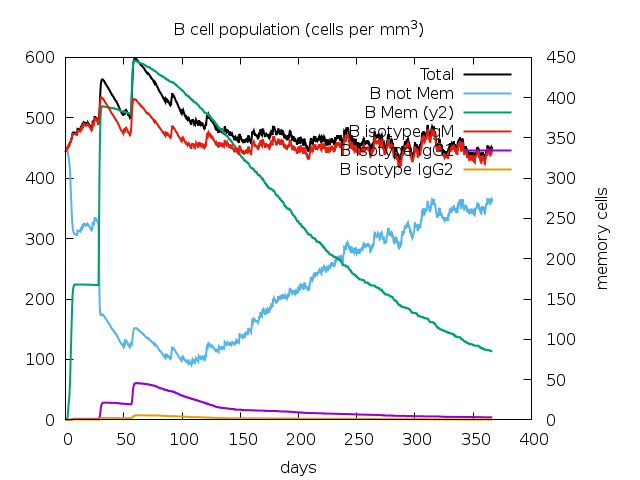
**

**C D**

**
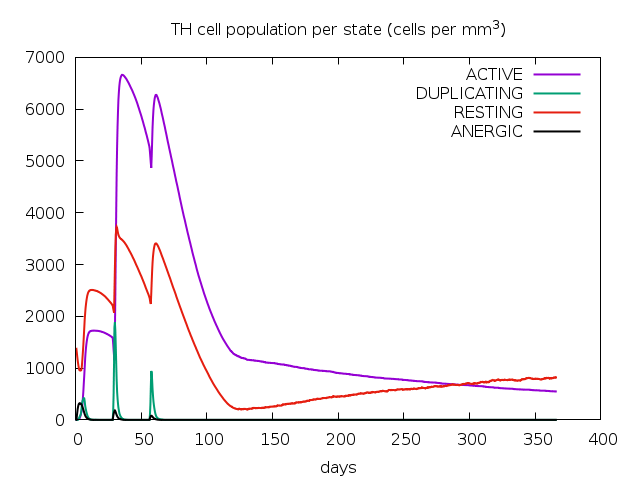

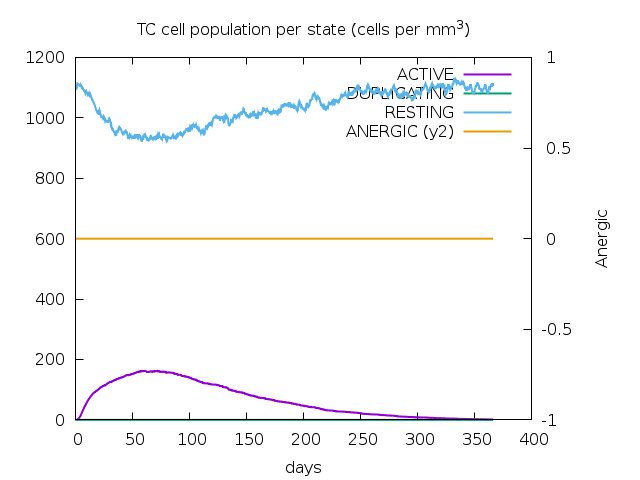
**

**Figure S4: SPVC_565 with adjuvants+HIS-tag:A)Antigen and immunoglobulin counts B)The changes observed in B-cell populations after given three injections, C) The development of T-helper, and D) T-cytotoxic cell populations per state after the injections**

**A B**

**
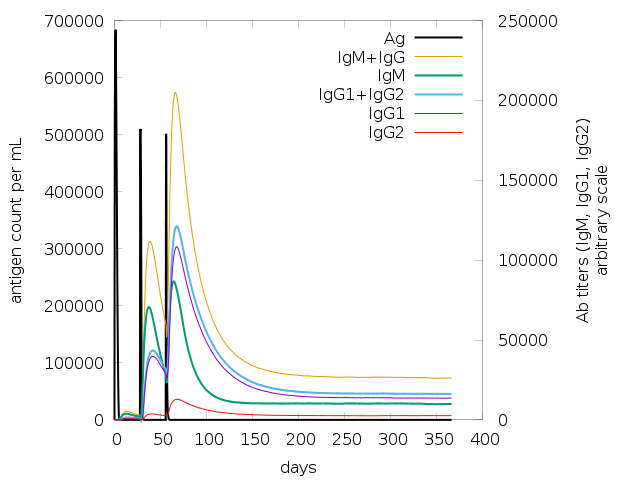

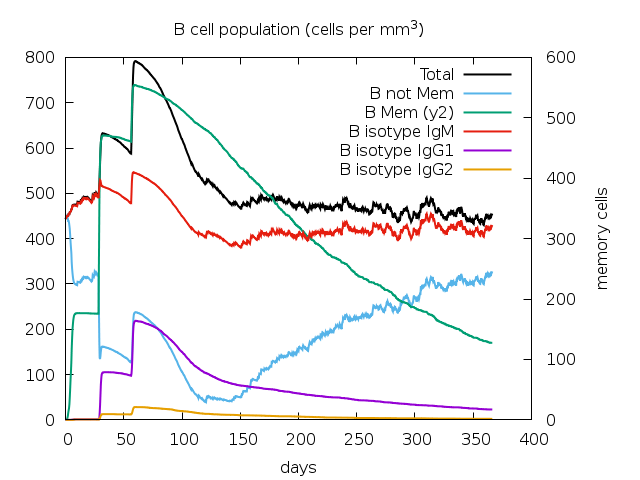
**

**D E**

**
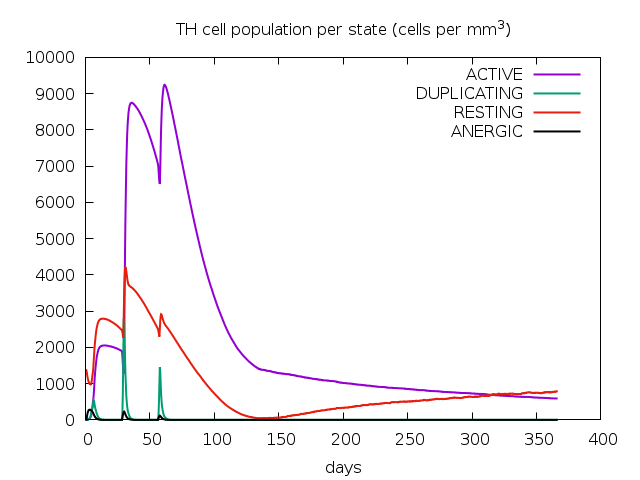

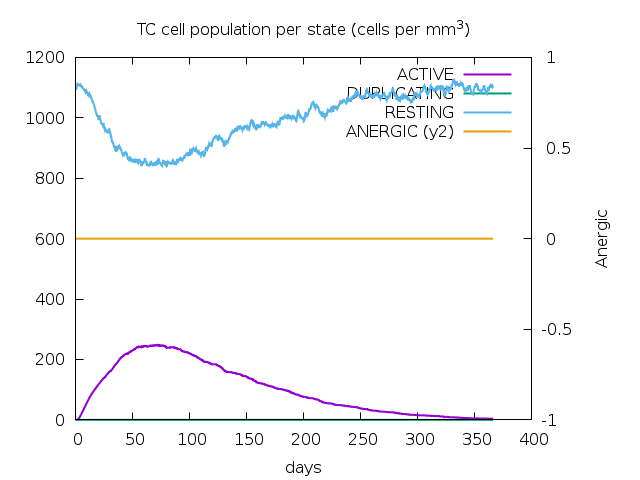
**

**Figure S5: SPVC_383 with adjuvants+HIS-tag:A)Antigen and immunoglobulin counts B)The changes observed in B-cell populations after given three injections, C) The development of T-helper, and D) T-cytotoxic cell populations per state after the injections**

**A B**

**C
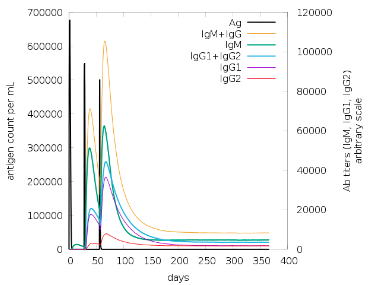
D
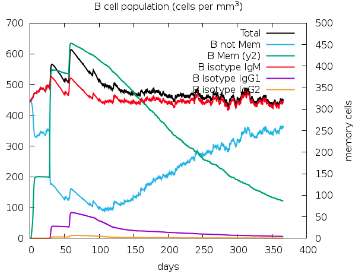
**

**
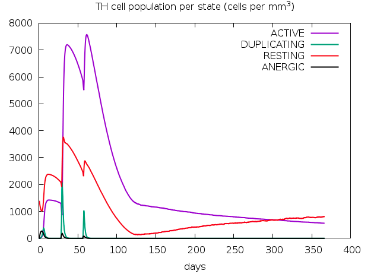

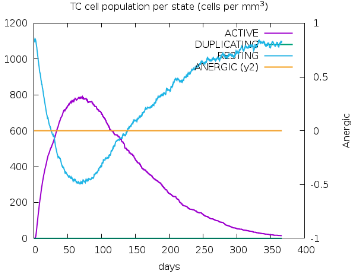
**

**Figure S6: SPVC_357 with adjuvants+HIS-tag:A)Antigen and immunoglobulin counts B)The changes observed in B-cell populations after given three injections, C) The development of T-helper, and D) T-cytotoxic cell populations per state after the injections**

**A B**

**
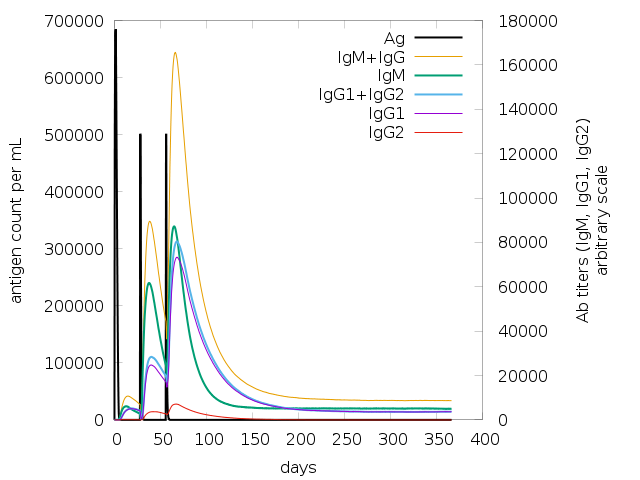

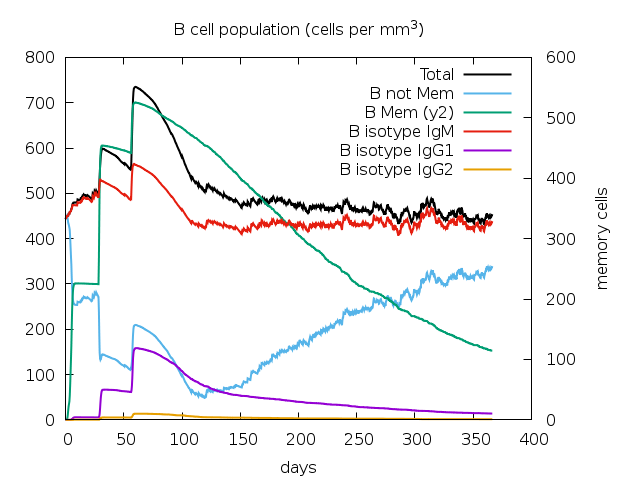
**

**C D**

**
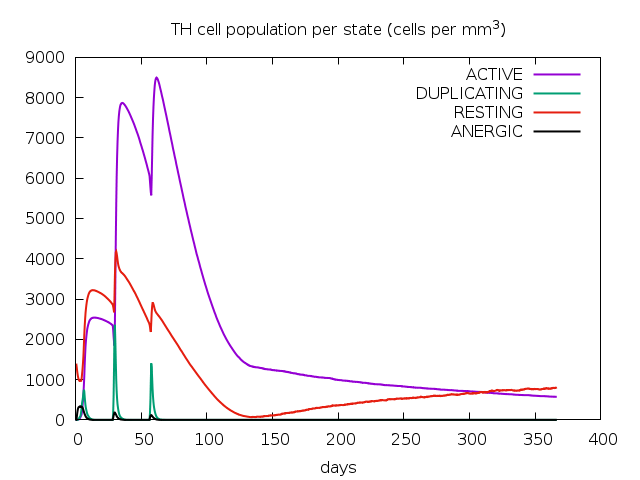

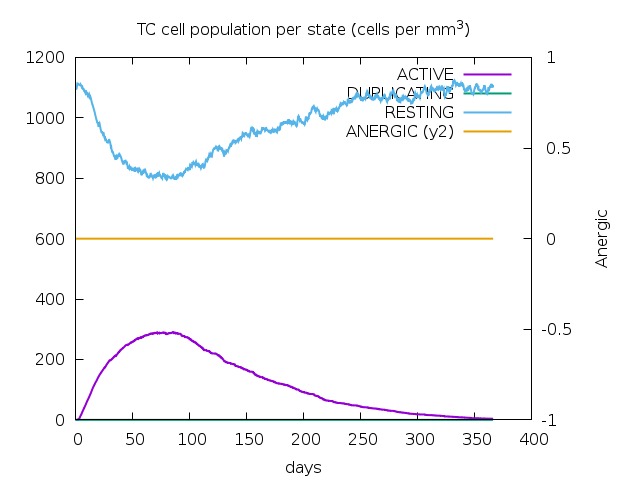
**

**Figure S7: SPVC_537 with adjuvants+HIS-tag:A)Antigen and immunoglobulin counts B)The changes observed in B-cell populations after given three injections, C) The development of T-helper, and D) T-cytotoxic cell populations per state after the injections**

**A B**


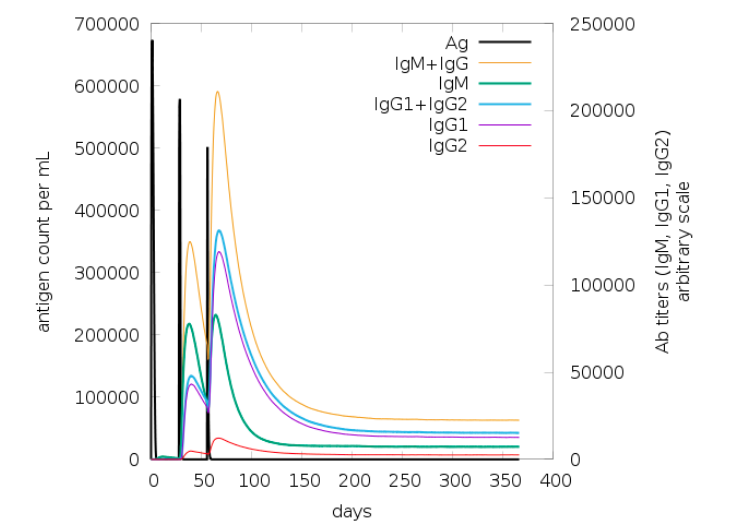

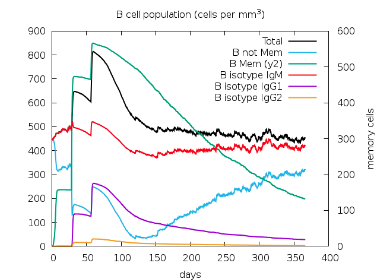


**C D**


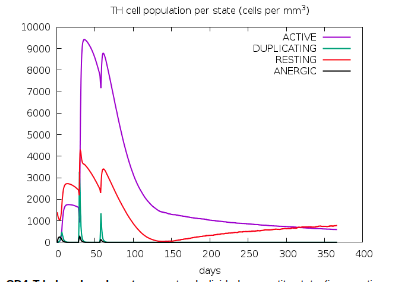

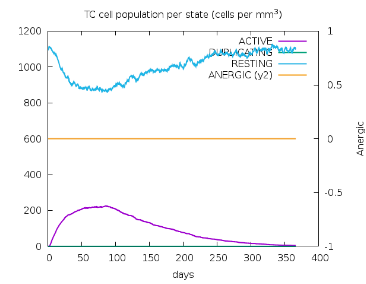


**Figure S8: REF_SEQ with adjuvants+HIS-tag: A)Antigen and immunoglobulin counts B)The changes observed in B-cell populations after given three injections, C) The development of T-helper, and D) T-cytotoxic cell populations per state after the injections**

**A B**

**
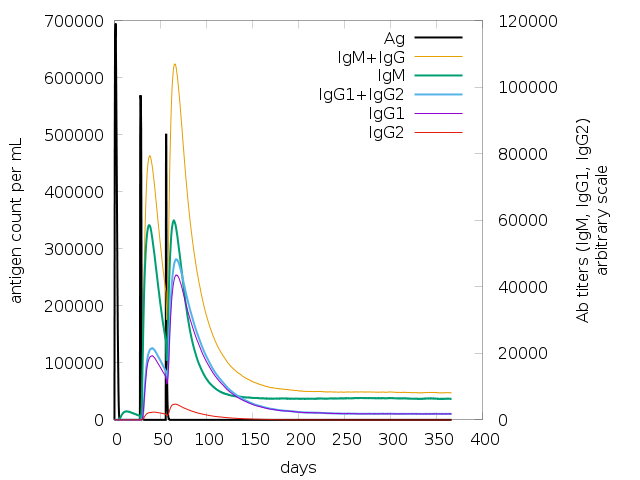

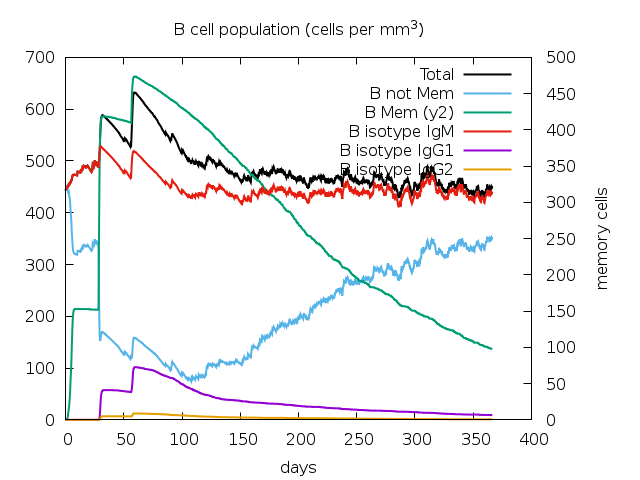
**

**C D**

**
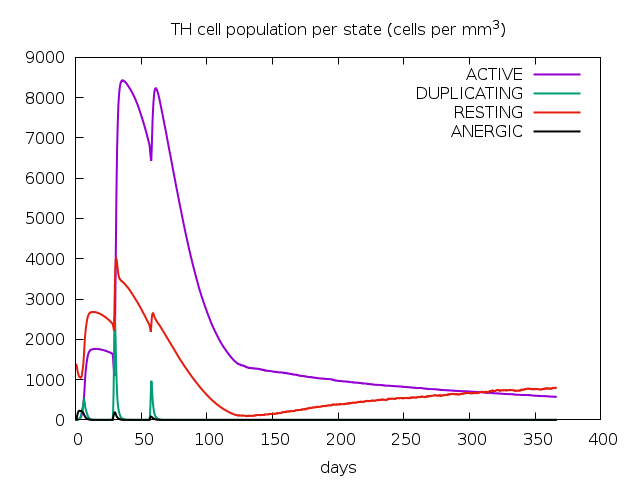

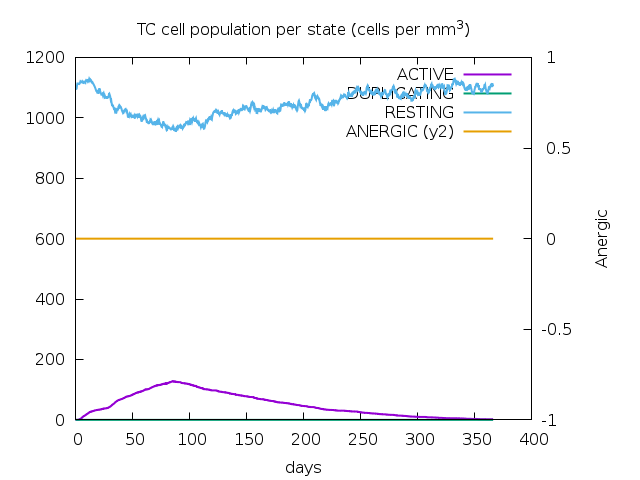
**

**Figure S9: SPVC_387 with adjuvants+HIS-tag:A)Antigen and immunoglobulin counts B)The changes observed in B-cell populations after given three injections, C) The development of T-helper, and D) T-cytotoxic cell populations per state after the injections**

**A B**

**
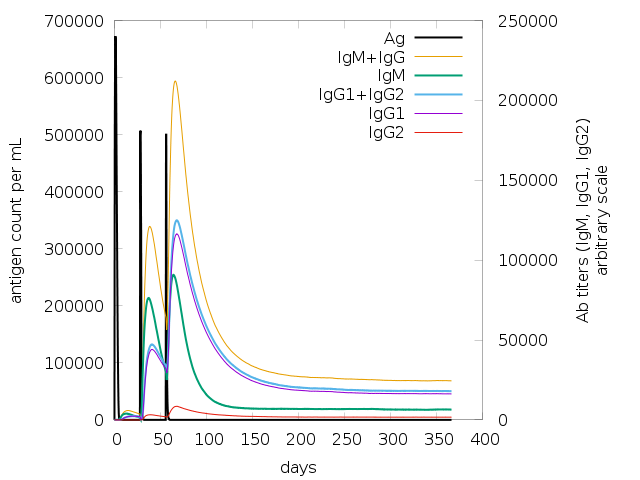

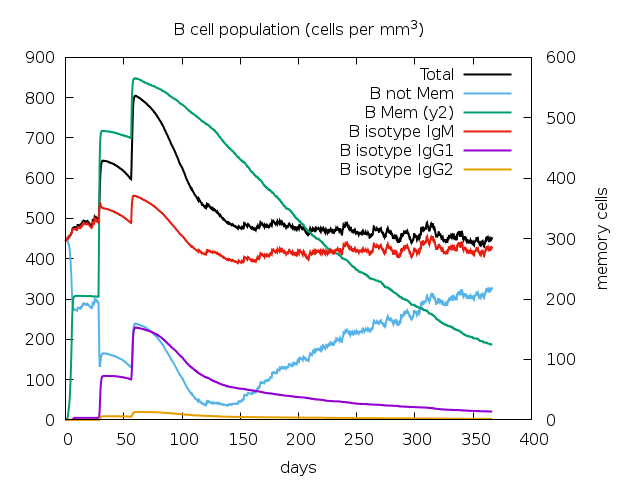
**

**C D**

**
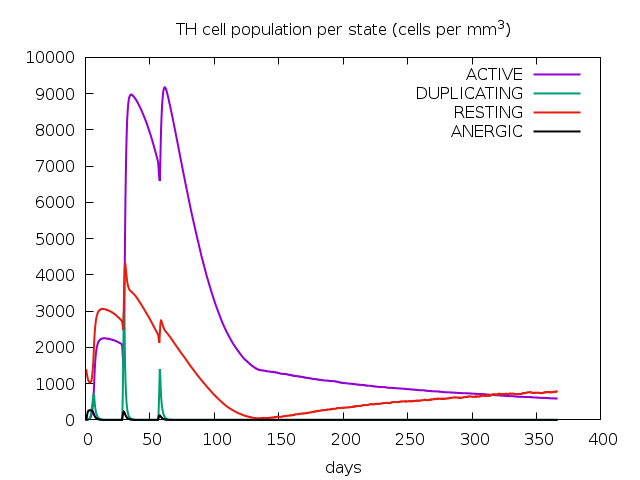

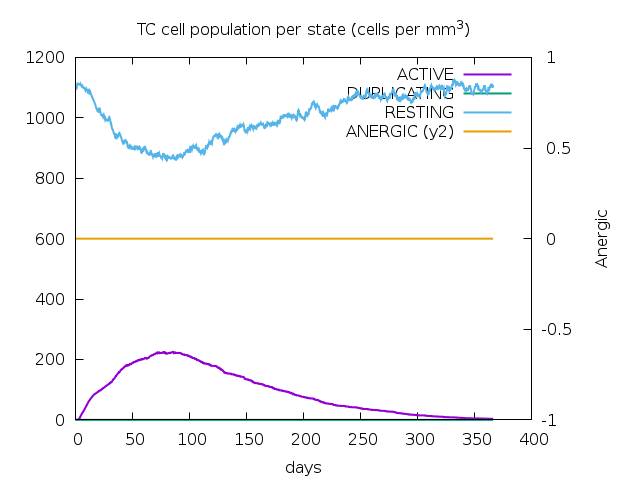
**

**Figure S10: SPVC_446 with adjuvants+HIS-tag:A)Antigen and immunoglobulin counts B)The changes observed in B-cell populations after given three injections, C) The development of T-helper, and D) T-cytotoxic cell populations per state after the injections**

**A B**

**
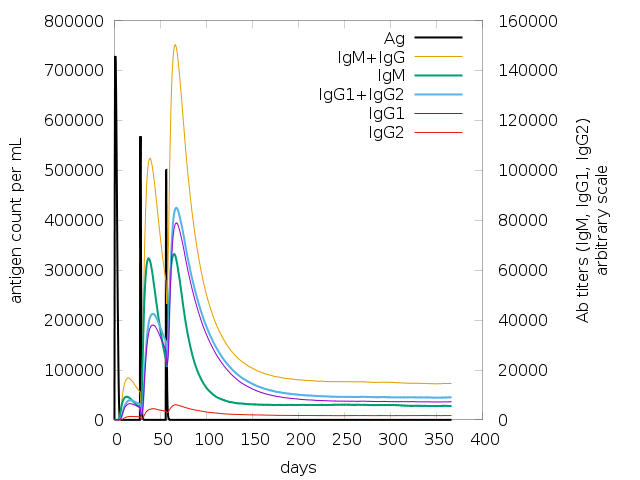

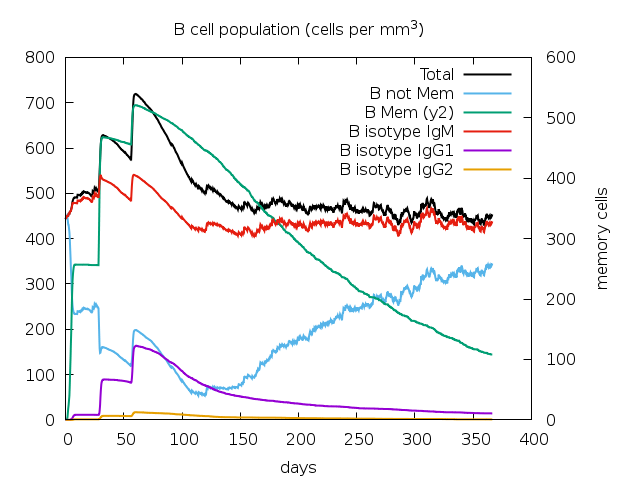
**

**C D**

**
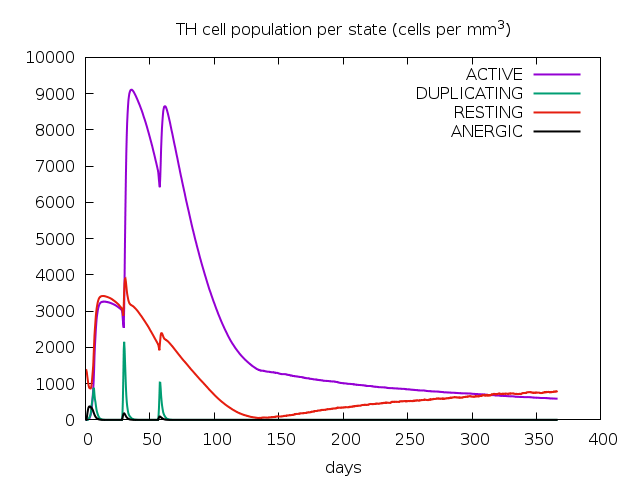

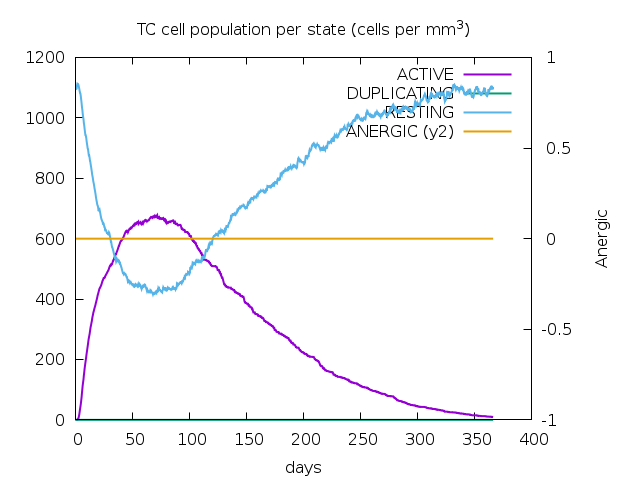
**

| **Figure S11: SPVC_214 without adjuvants+HIS-tag:A)Antigen and immunoglobulin counts B)The changes observed in B-cell populations after given three injections, C) The development of T-helper, and D) T-cytotoxic cell populations per state after the injections**  **A B**  **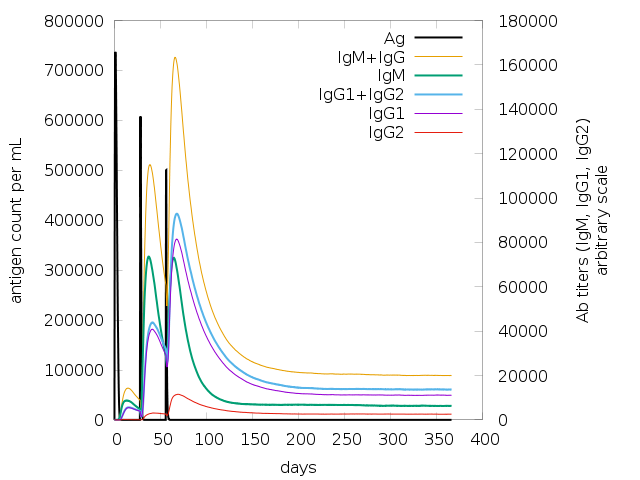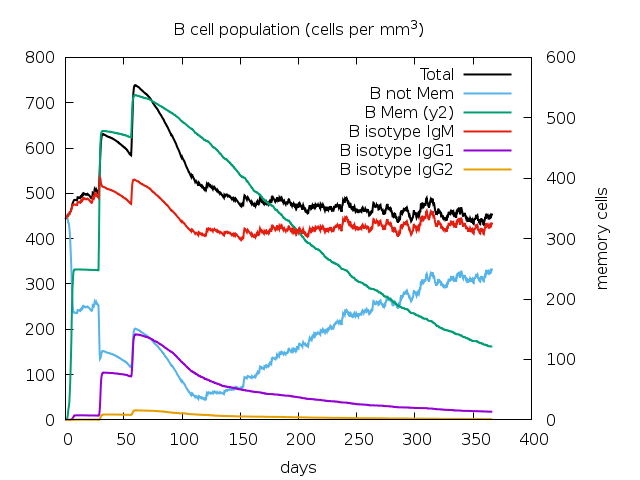**  **C D**  **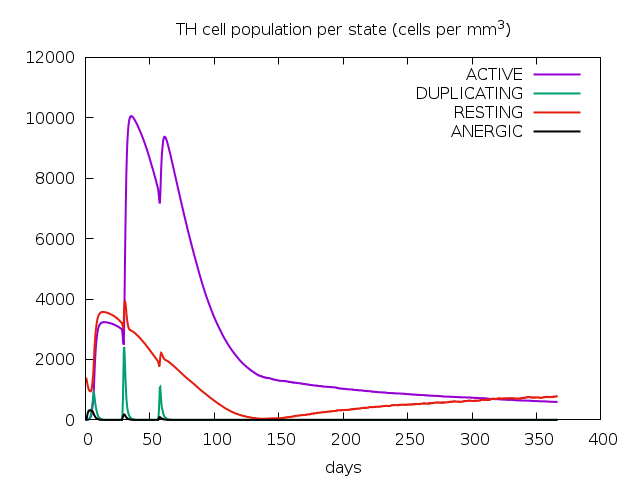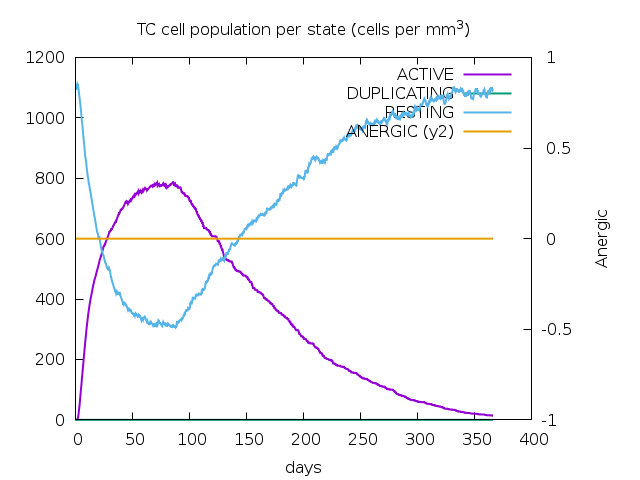** |
| --- |
| **Figure S12: SPVC_32 without adjuvants+HIS-tag:A)Antigen and immunoglobulin counts B)The changes observed in B-cell populations after given three injections, C) The development of T-helper, and D) T-cytotoxic cell populations per state after the injections** |
|  |
|  |

**A B**

**
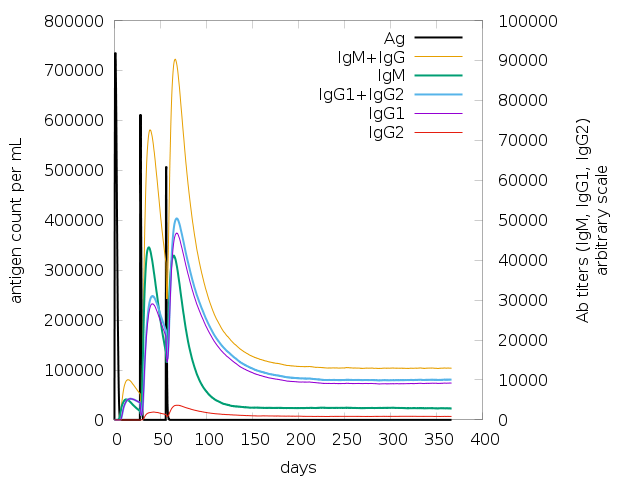

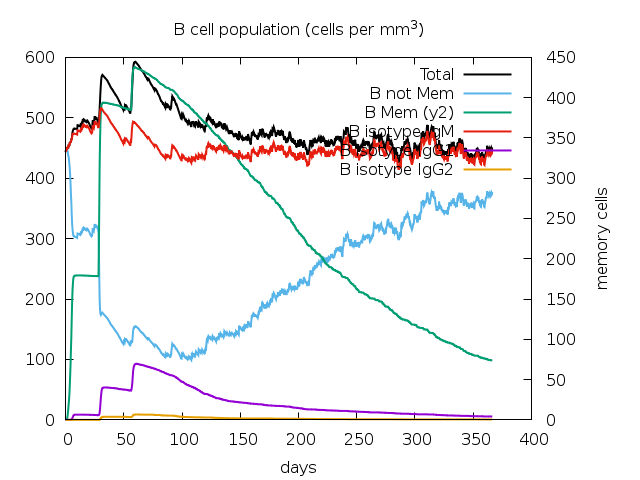
**

**C D**

**
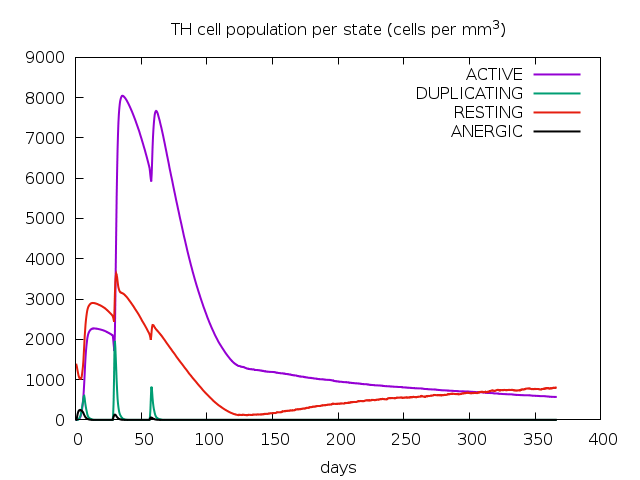

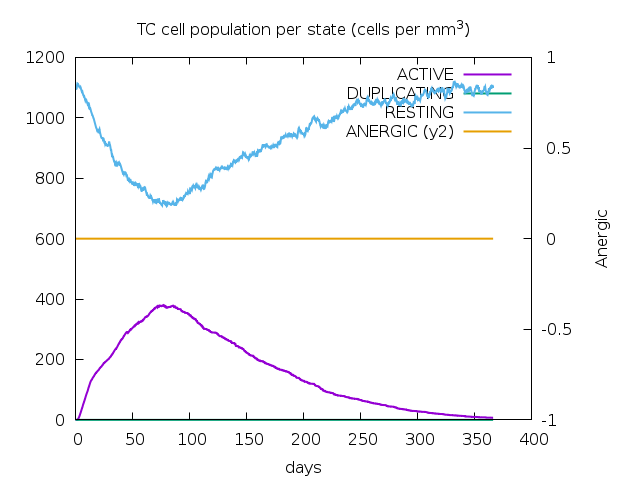
**

**Figure S13: SPVC_206 without adjuvants+HIS-tag:A) Antigen and immunoglobulin counts B)The changes observed in B-cell populations after given three injections, C) The development of T-helper, and D) T-cytotoxic cell populations per state after the injections**

**A B**

**
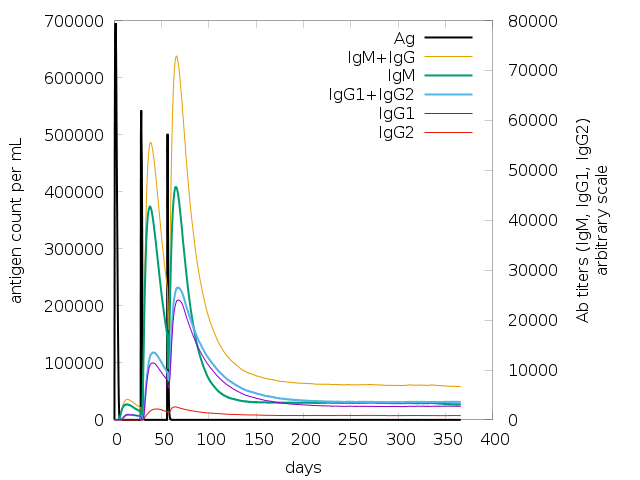

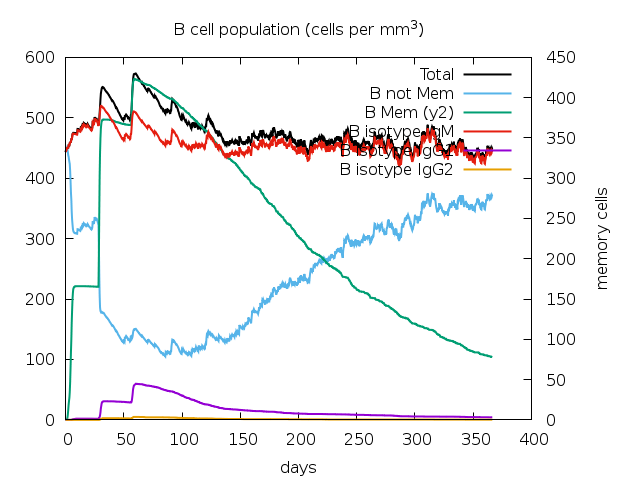
**

**C D**

**
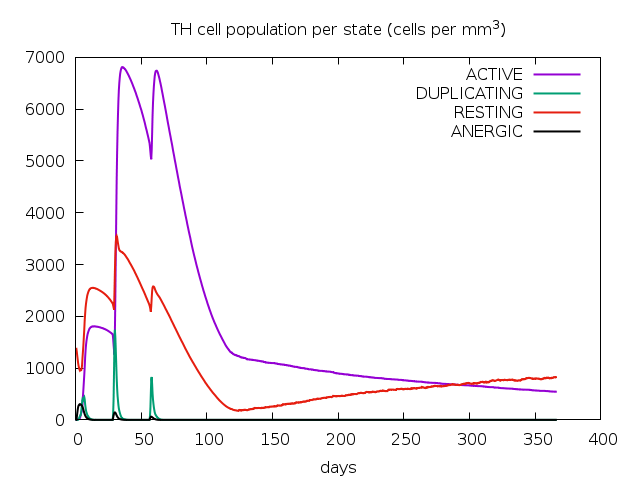

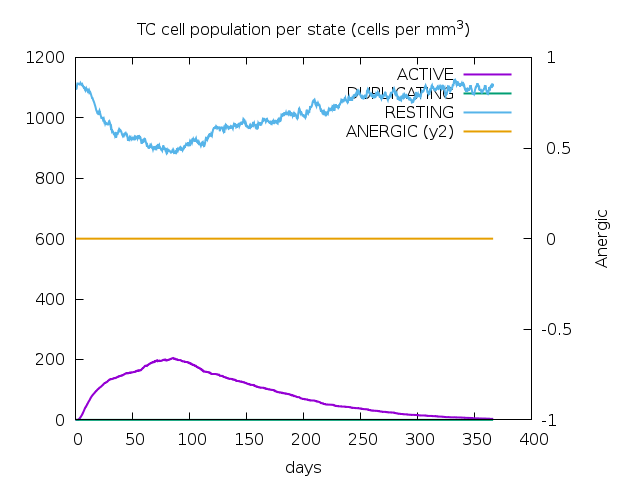
**

**Figure S14: SPVC_565 without adjuvants+HIS-tag:A)Antigen and immunoglobulin counts B)The changes observed in B-cell populations after given three injections, C) The development of T-helper, and D) T-cytotoxic cell populations per state after the injections**

**A B**

**
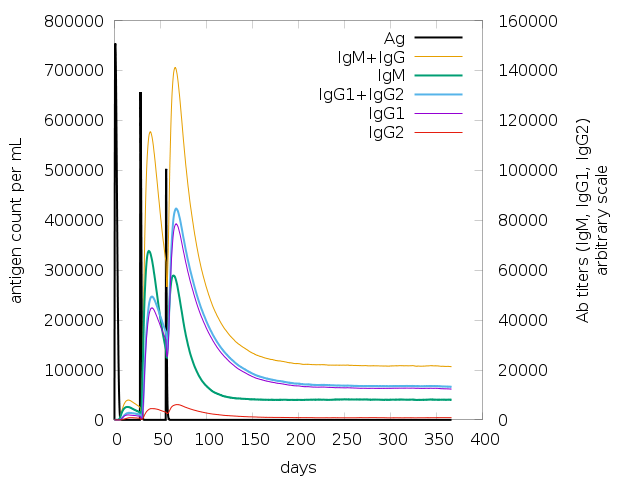

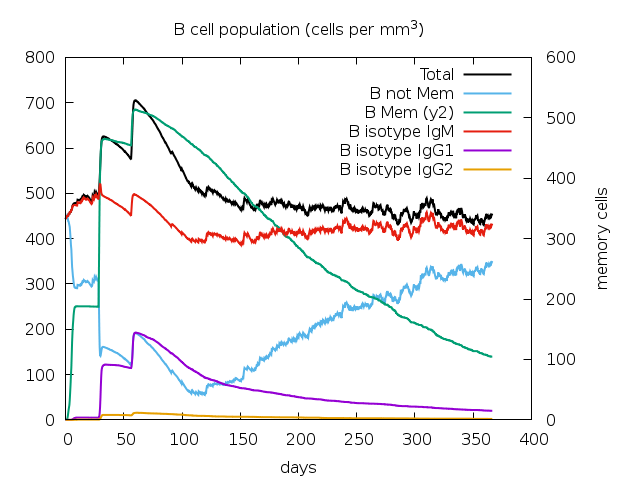
**

**C D**

**
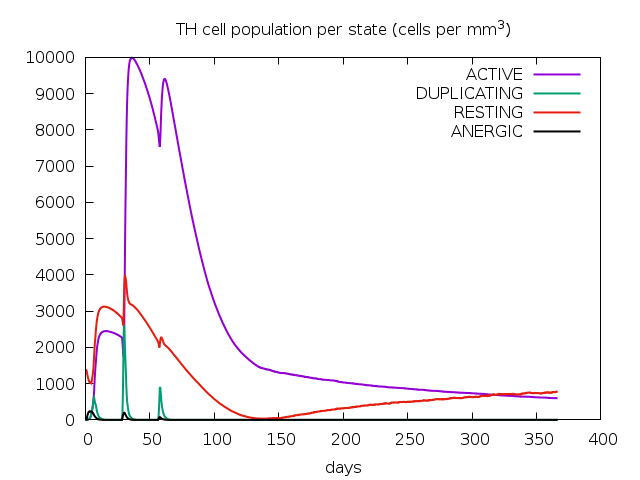

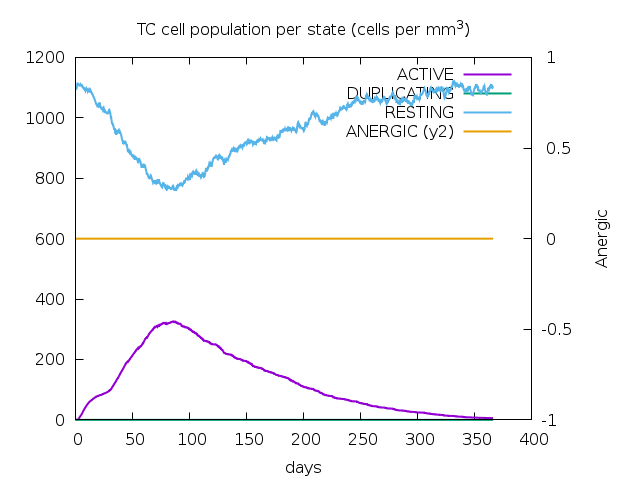
**

**Figure S15: SPVC_383 without adjuvants+HIS-tag:A)Antigen and immunoglobulin counts B)The changes observed in B-cell populations after given three injections, C) The development of T-helper, and D) T-cytotoxic cell populations per state after the injections**

**A B**

**
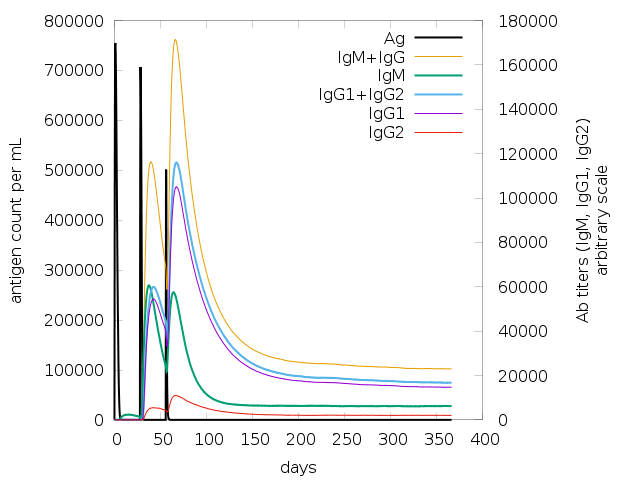

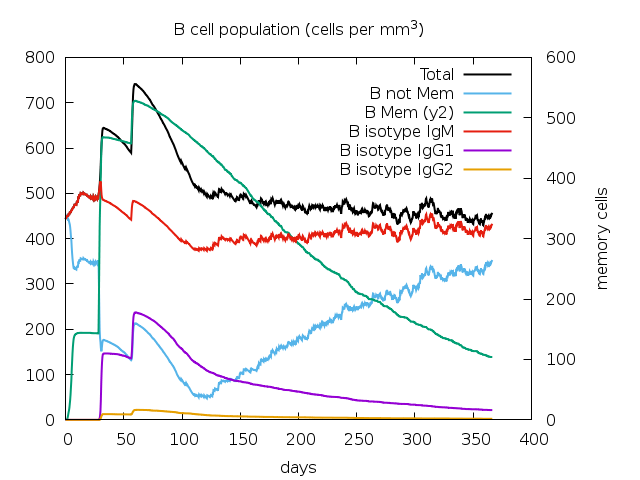
**

**C D**

**
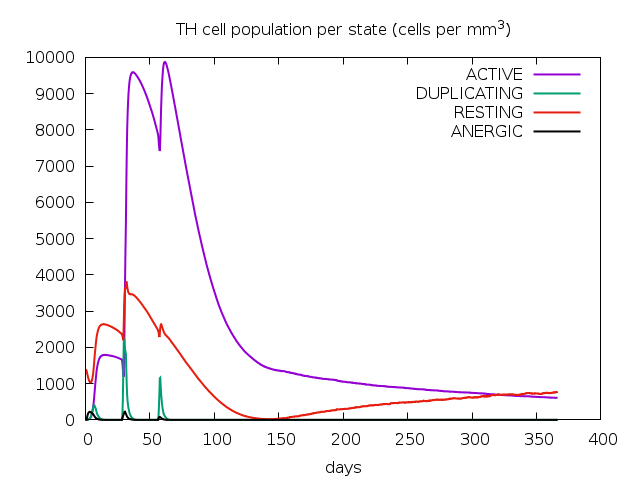

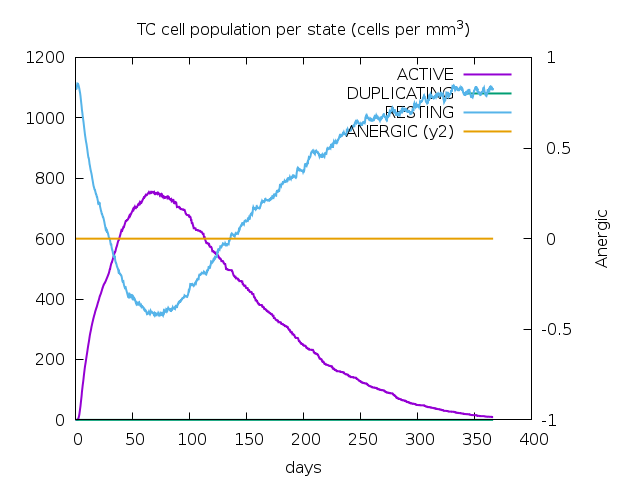
**

**Figure S16: SPVC_357 without adjuvants+HIS-tag:A)Antigen and immunoglobulin counts B)The changes observed in B-cell populations after given three injections, C) The development of T-helper, and D) T-cytotoxic cell populations per state after the injections**

**A B**

**
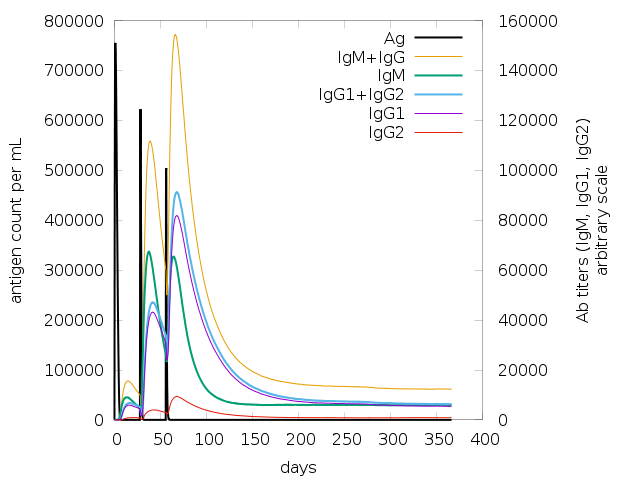

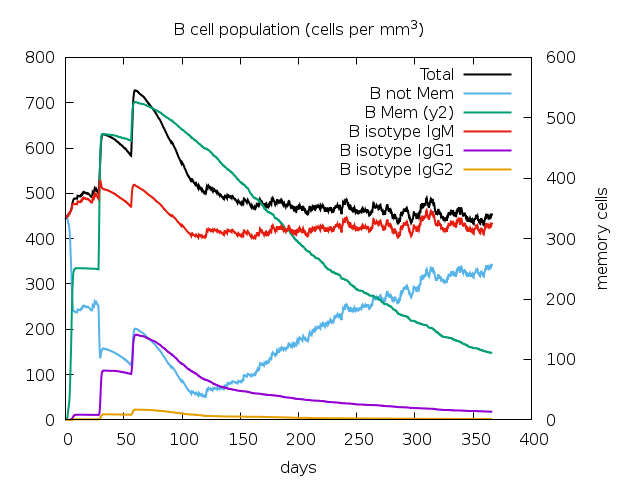
**

**C D**

**
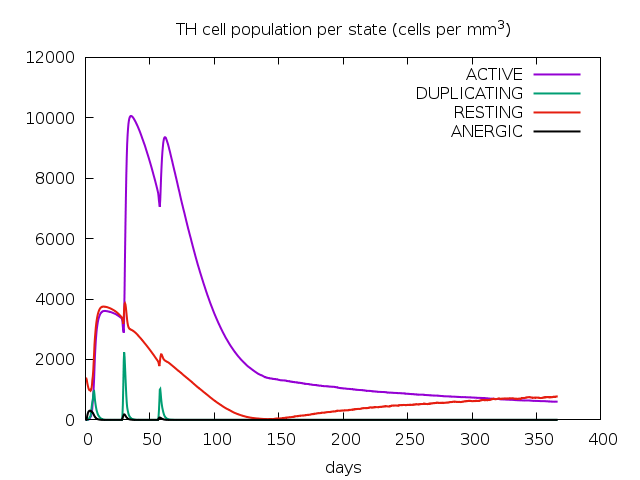

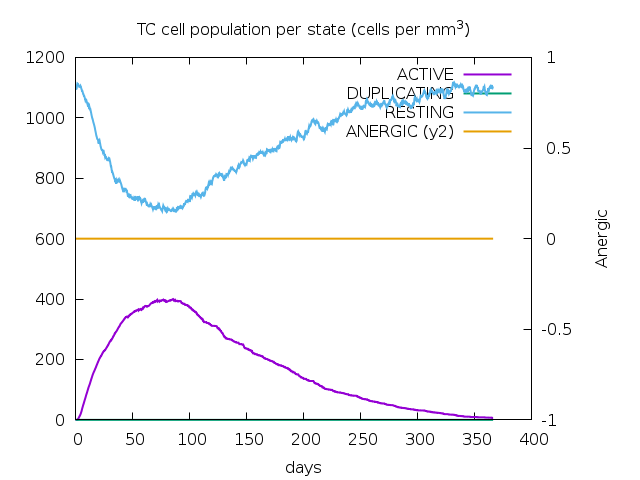
**

**Figure S17: SPVC_537 without adjuvants+HIS-tag:A)Antigen and immunoglobulin counts B)The changes observed in B-cell populations after given three injections, C) The development of T-helper, and D) T-cytotoxic cell populations per state after the injections**

**A B**

**
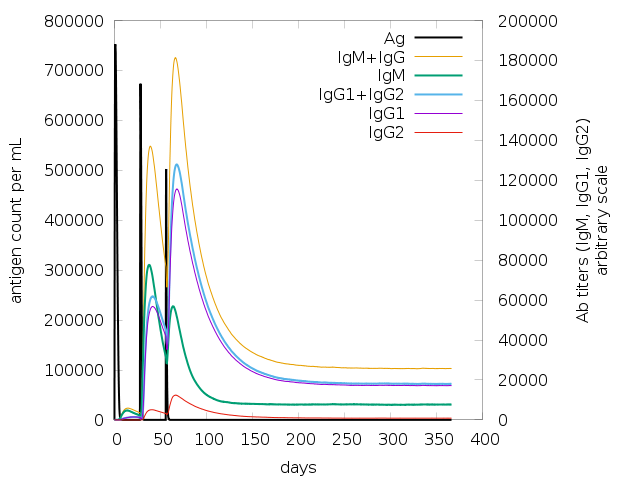

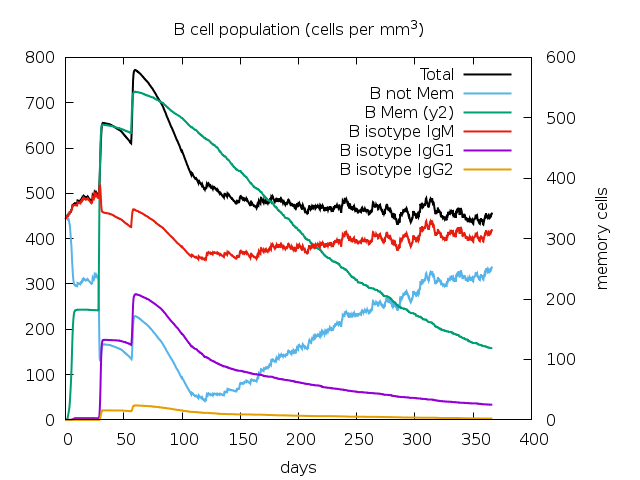
**

**C D**

**
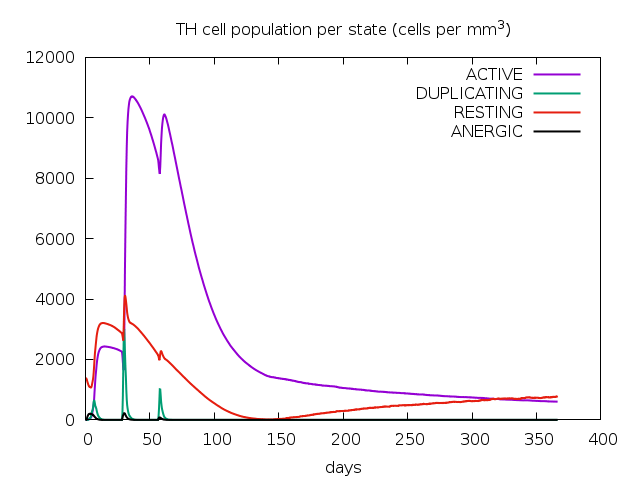

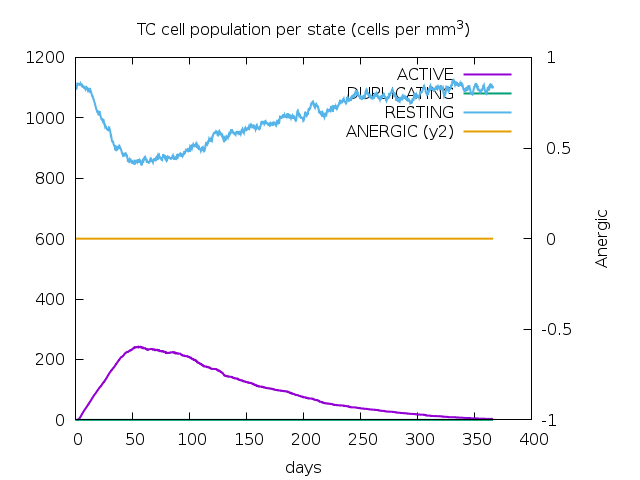
**

**Figure S18: REF_SEQ without adjuvants+HIS-tag:A)Antigen and immunoglobulin counts B)The changes observed in B-cell populations after given three injections, C) The development of T-helper, and D) T-cytotoxic cell populations per state after the injections**

**A B**

**
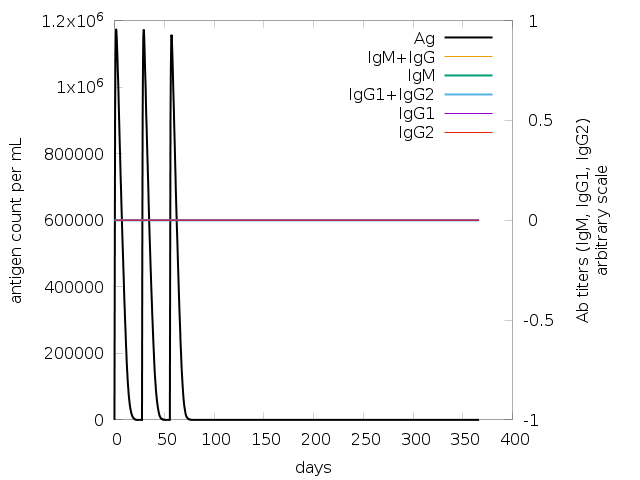

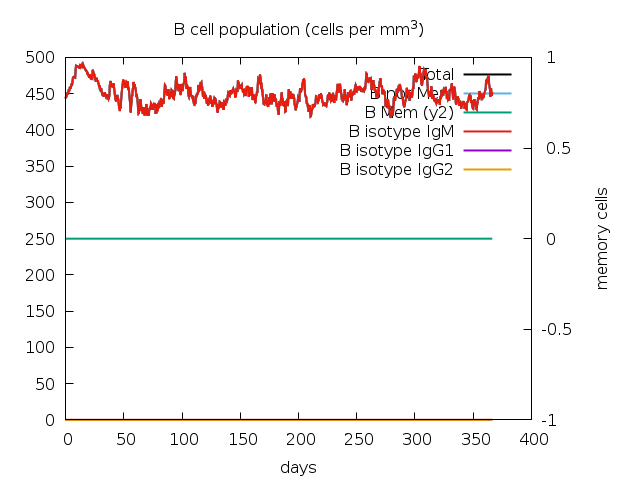
**

**C D**

**
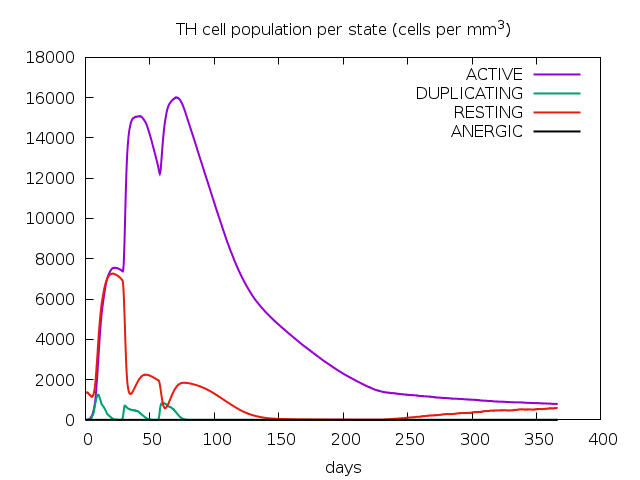

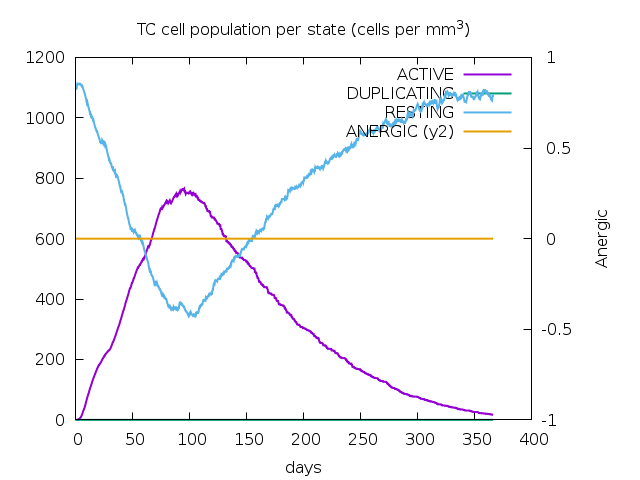
**

**Figure S19: SPVC_387 without adjuvants+HIS-tag:A)Antigen and immunoglobulin counts B)The changes observed in B-cell populations after given three injections, C) The development of T-helper, and D) T-cytotoxic cell populations per state after the injections**

**A B**

**
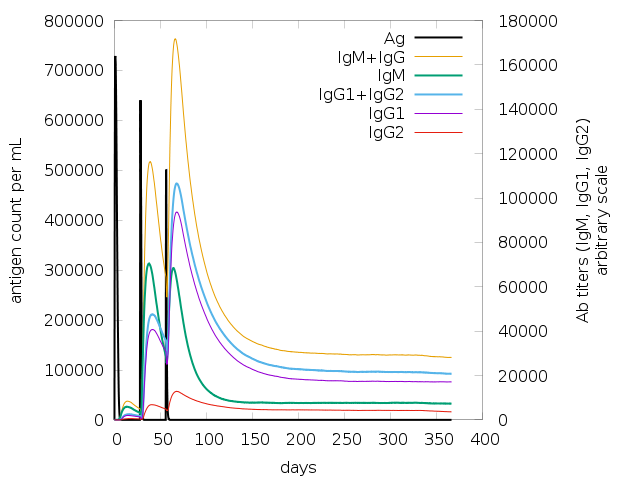

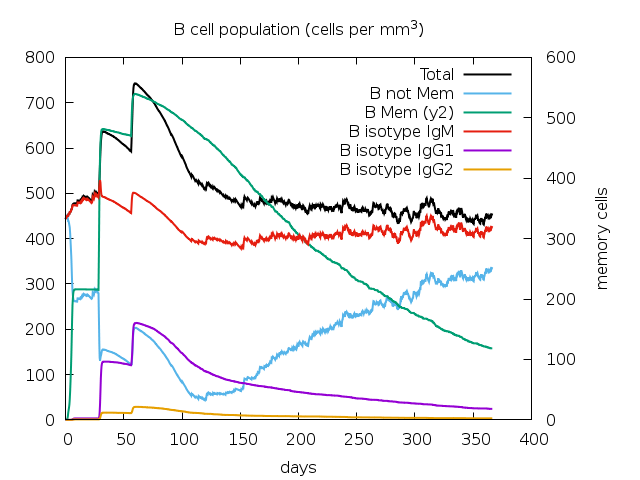
**

**C D**

**
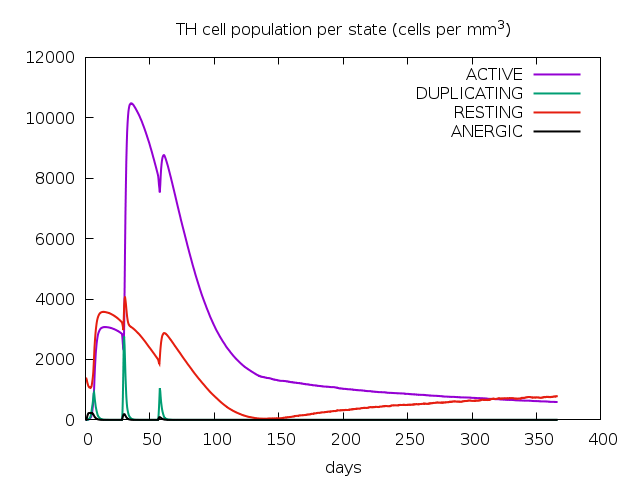

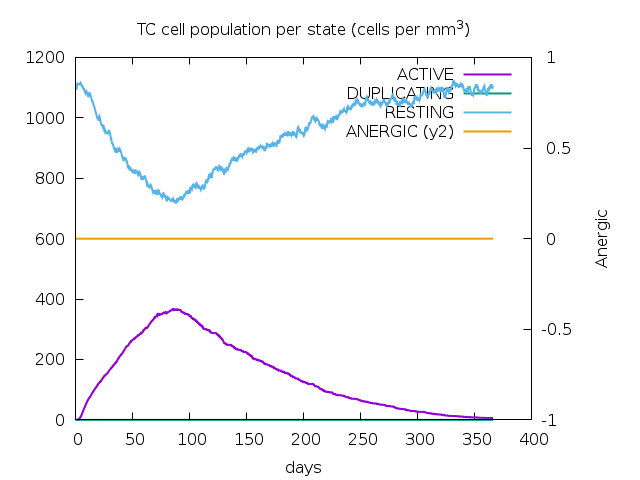
**

**Figure S20: SPVC_446 without adjuvants+HIS-tag:A)Antigen and immunoglobulin counts B)The changes observed in B-cell populations after given three injections, C) The development of T-helper, and D) T-cytotoxic cell populations per state after the injections**
