# Supplementary material for: An in silico approach to study the role of epitope order in the multi-epitope-based peptide (MEBP) vaccine design
Source: Sci Rep. 2022 Jul 22;12:12584. doi: 10.1038/s41598-022-16445-3 (PMC9307121; doi:10.1038/s41598-022-16445-3)
Supplement: Supplementary file 3 — Supplementary Information 2. [file 41598_2022_16445_MOESM3_ESM.docx]

**An *in silico* approach to study the role of epitope order in the multi-epitope-based peptide (MEBP) vaccine design**

**Muthu Raj Salaikumaran^1^, Prasanna Sudharson Kasamuthu^1^, Veeranarayanan Surya Aathmanathan^1^, Burra V L S Prasad^1^***

^1^Centre for Advanced Research and Innovation in Structural Biology of Diseases, K L E F University, Vaddeswaram, Andhra Pradesh 522 502, India

^*^Corresponding Author: [dr.prasad.bvls@gmail.com](mailto:dr.prasad.bvls@gmail.com)

**Supplementary Material 3 (Normalized Values)**

| **TLR4** | | | | | | | | | | | | |
| --- | --- | --- | --- | --- | --- | --- | --- | --- | --- | --- | --- | --- |
| **MEBP Variant ID** | **Stability** | **Accessibility** | **Solubility** | **Disorder** | **Aggregation** | **Hydrophobicity** | **Antigenicity** | **Z RANK SCORE** | **MMGBSA/PBSA score ΔG (Kcal/mol)** | **Max. RMSD of Vaccine complex(nm)** | **Max. RMSF of Vaccine complex (nm)** | **RMVP** |
| **REF_SEQ** | 0.997 | 1.003 | 1.011 | 1.049 | 1.138 | 0.935 | 0.937 | 0.980 | 0.951 | 0.167 | 0.527 | 3.933 |
| **SPVC_206** | 0.982 | 0.990 | 1.011 | 1.049 | 0.966 | 1.028 | 0.982 | 0.904 | 1.080 | 0.129 | 0.519 | 4.313 |
| **SPVC_214** | 0.979 | 1.009 | 1.011 | 0.926 | 0.966 | 0.888 | 0.937 | 1.064 | 0.676 | 1.612 | 2.694 | 0.366 |
| **SPVC_32** | 1.015 | 0.990 | 1.023 | 0.988 | 0.931 | 0.981 | 0.997 | 0.890 | 1.207 | 0.141 | 0.564 | 4.479 |
| **SPVC_357** | 0.988 | 0.987 | 1.011 | 1.049 | 0.966 | 1.028 | 0.952 | 0.919 | 0.967 | 0.185 | 0.738 | 3.914 |
| **SPVC_383** | 0.988 | 1.027 | 1.011 | 0.988 | 0.966 | 1.028 | 0.982 | 0.939 | 0.922 | 0.132 | 0.458 | 4.353 |
| **SPVC_387** | 0.988 | 0.983 | 0.999 | 0.926 | 1.138 | 1.028 | 1.012 | 1.049 | 1.013 | 0.151 | 0.738 | 4.119 |
| **SPVC_446** | 1.018 | 1.009 | 0.900 | 0.988 | 1.069 | 1.121 | 1.178 | 0.971 | 1.090 | 0.151 | 0.438 | 4.642 |
| **SPVC_537** | 1.030 | 1.016 | 1.011 | 0.988 | 0.931 | 0.981 | 1.027 | 1.091 | 1.129 | 0.135 | 0.703 | 4.529 |
| **SPVC_565** | 1.015 | 0.987 | 1.011 | 1.049 | 0.931 | 0.981 | 0.997 | 1.193 | 0.964 | 1.398 | 2.617 | 1.153 |

| **TLR8** | | | | | | | | | | | | |
| --- | --- | --- | --- | --- | --- | --- | --- | --- | --- | --- | --- | --- |
| **MEBP Variant ID** | **Stability** | **Accessibility** | **Solubility** | **Disorder** | **Aggregation** | **Hydrophobicity** | **Antigenicity** | **Z RANK SCORE** | **MMGBSA/PBSA score ΔG (Kcal/mol)** | **Max. RMSD of Vaccine complex(nm)** | **Max. RMSF of Vaccine complex (nm)** | **RMVP** |
| **REF_SEQ** | 0.997 | 1.003 | 1.011 | 1.049 | 1.138 | 0.935 | 0.937 | 1.055 | 1.829 | 0.239 | 0.678 | 4.662 |
| **SPVC_206** | 0.982 | 0.990 | 1.011 | 1.049 | 0.966 | 1.028 | 0.982 | 1.017 | 0.840 | 0.150 | 0.640 | 4.044 |
| **SPVC_214** | 0.979 | 1.009 | 1.011 | 0.926 | 0.966 | 0.888 | 0.937 | 1.020 | 1.125 | 0.212 | 0.869 | 3.995 |
| **SPVC_32** | 1.015 | 0.990 | 1.023 | 0.988 | 0.931 | 0.981 | 0.997 | 0.956 | 0.603 | 0.301 | 0.888 | 3.457 |
| **SPVC_357** | 0.988 | 0.987 | 1.011 | 1.049 | 0.966 | 1.028 | 0.952 | 0.949 | 0.562 | 0.324 | 1.223 | 2.916 |
| **SPVC_383** | 0.988 | 1.027 | 1.011 | 0.988 | 0.966 | 1.028 | 0.982 | 0.908 | 0.977 | 1.535 | 2.235 | 1.197 |
| **SPVC_387** | 0.988 | 0.983 | 0.999 | 0.926 | 1.138 | 1.028 | 1.012 | 1.052 | 1.264 | 1.624 | 1.194 | 2.444 |
| **SPVC_446** | 1.018 | 1.009 | 0.900 | 0.988 | 1.069 | 1.121 | 1.178 | 0.943 | 0.882 | 0.139 | 0.640 | 4.216 |
| **SPVC_537** | 1.030 | 1.016 | 1.011 | 0.988 | 0.931 | 0.981 | 1.027 | 1.186 | 0.880 | 0.189 | 0.654 | 4.371 |
| **SPVC_565** | 1.015 | 0.987 | 1.011 | 1.049 | 0.931 | 0.981 | 0.997 | 0.914 | 1.038 | 0.174 | 0.984 | 3.806 |
